# Pan-Cancer Analyses Reveal Oncogenic and Immunological Role of PLOD2

**DOI:** 10.3389/fgene.2022.864655

**Published:** 2022-05-02

**Authors:** Qiqi Xu, Na Kong, Yiguo Zhao, Quan Wu, Xin Wang, Xiaodong Xun, Pengji Gao

**Affiliations:** General Surgery of Beijing Jishuitan Hospital, The Fourth Clinical Medical College of Peking University, Beijing, China

**Keywords:** PLOD2, prognostic biomarker, pan-cancer analysis, immune infiltration, cancer immunity

## Abstract

Some previous studies have shown that *PLOD2* has some value in tumorigenesis. However, the broad significance of *PLOD2* has not been discussed in depth. This study was aimed at elaborated and summarized the value of PLOD2 in various tumors. First, we integrated GTEx, The Cancer Genome Atlas and Cancer Cell Line Encyclopedia databases to analyze the expression of *PLOD2*, and found that it was expressed differently in normal tissues and significantly highly expressed in most tumors compared with normal tissues. Second, our analysis revealed that *PLOD2* expression was negatively correlated with the prognosis of several tumors. For gastric cancer, the median overall survival time was significantly higher in the *PLOD2* low expression group [HR 0.616 (95%CI 0.442–0.858), *p =* 0.004]. Third, for tumor immunity, *PLOD2* was significantly associated with tumor infiltration, including immune infiltrating cells; immune checkpoint expression; immune microenvironment scores (immune score, stromal score and estimate scores); immunotherapy-related scores (tumor mutational burden, microsatellite instability, tumor neoantigen burden); expression of DNA repair genes Mismatch Repairs and methyltransferase; and enrichment analyses identified *PLOD2*-associated terms and pathways. Lastly, twenty pairs of gastric cancer and adjacent immunohistochemistry showed that *PLOD2* was significantly overexpressed in gastric cancer (*p <* 0.001). Collectively, *PLOD2* played a significant role in tumorigenesis and maybe serve as a potential biomarker for diagnosis and prognosis in cancers.

## Introduction

The incidence of malignant neoplasms has increased at an alarming rate over the past few decades (Bray et al., 2021; Sung et al., 2021). Pan-cancer analysis aims to examine the similarities and differences between genomic and cellular changes found in different tumor types (Chang et al., 2013; Gentles et al., 2015). Pan-cancer analysis projects, such as the Cancer Cell Line Encyclopedia (CCLE) and The Cancer Genome Atlas (TCGA), were created based on the evaluation of different human cancer cell lines and tissues at the epigenomic, genomic, proteomic, and transcriptomic levels. TCGA provides medical researchers with irreplaceable genomic, epigenomic, transcriptomic, and clinical data (Hutter and Zenklusen, 2018). What’s more, it has boosted the study of tumor immunology and immunotherapy (Thorsson et al., 2018). Pan-cancer analysis has made an important contribution to the development of life science and medicine. For example, on 4 February 2020, Pan-Cancer Analysis of Whole Genomes (PCAWG) Consortium of the International Cancer Genome Consortium (ICGC) and TCGA published six articles in Nature (Author Anonyms, 2020), proposing the most comprehensive cancer genome analysis so far. Different from the previous focus on protein coding regions, this time is to analyze the whole genome of cancer. This program covers six aspects: pan-cancer analysis of whole genomes (ICGC/TCGA Pan-Cancer Analysis of Whole Genomes Consortium, 2020); analyses of non-coding somatic drivers in 2,658 cancer whole genomes (Rheinbay et al., 2020); the repertoire of mutational signatures in human cancer (Alexandrov et al., 2020); patterns of somatic structural variation in human cancer genomes (Li Y et al., 2020); the evolutionary history of 2,658 cancers (Gerstung et al., 2020); genomic basis for RNA alterations in cancer (Calabrese et al., 2020).

*PLOD2* (Procollagen-Lysine,2-Oxoglutarate 5-Dioxygenase 2) was a member of PLOD family (*PLOD1*, *PLOD2*, *PLOD3*), which encodes a special protein (also known as LH2, TLH2 and BRKS2) mediating the formation of stabilized collagen cross-links (Genecards, 2021). Collagen crosslinking played a key role in extracellular matrix (Du et al., 2017). Various studies have shown the extracellular matrix (ECM) to be closely to tumor cell growth and metastasis (Gilkes et al., 2014; Tadeo et al., 2016). Upregulation of *PLOD2* has been observed in several malignancies such as bladder cancer (Miyamoto et al., 2016), lung cancer (Kocher et al., 2021), gastric cancer (Song et al., 2021), head and neck squamous cell cancer (Xin et al., 2021), breast cancer (Gilkes et al., 2013), etc. Kiyozumi et al. showed that PLOD2 was significantly associated with peritoneal dissemination in gastric cancer (Kiyozumi et al., 2018). In the metastatic group, PLOD2 was significantly highly expressed, both at the mRNA and protein level. Silencing PLOD2 significantly reduced cell invasiveness and migration *in vitro*. Further experiments showed that this was mainly regulated by HIF-1a in hypoxia condition. Another study on PLOD2 and 5-FU resistance in gastric cancer showed that PLOD2 could enhance 5-FU resistance by regulating BCRP and inhibit cell apoptosis by affecting the expression of Bax and Bcl2 (Wang et al., 2020). Downregulation of PLOD2 facilitated the sensitivity of gastric cancer to 5-FU *in vivo*. Generally, *PLOD2* plays an important role in both tumor growth and closely related to the prognosis of patients. Although a number of studies have been carried out on *PLOD2*, no single study exists which could overall evaluate its effects on considerable types of cancers. To understand the functions (especially cancer immunity) of *PLOD2* in different tumors, a comprehensive pan-cancer analysis was necessary.

To that end, we will elucidate the expression of *PLOD2* in 33 different malignant tumors in the following aspects and focus on gastric cancer. All in all, the results of our study provide information regarding the role of *PLOD2* in tumors, reveal the relationship between *PLOD2* and tumor-immune interactions, and clarify the potential underlying mechanisms.

## Materials and Methods

### Patient Datasets and Processes

This study has been approved by the Ethics Committee of Beijing Jishuitan Hospital (202004-58). First, we analyzed the *PLOD2* gene expression levels in each normal tissue using the GTEx (Genotype-Tissue Expression) database (https://xena.ucsc.edu/). Second, the data of each tumor cell line were downloaded from the CCLE database (https://sites.broadinstitute.org/ccle), and the expression levels of 21 tissues were analyzed according to the tissue source. Third, we obtained gene expression differences between cancer and para-cancer tissues in individual tumor samples from the TCGA database (https://portal.gdc.cancer.gov/). Fourth, considering the small number of normal samples in TCGA database, we integrated data from GTEx and TCGA database to analyze the differences expression in multiple tumors.

### Procollagen-Lysine,2-Oxoglutarate 5-Dioxygenase 2 Expression and its Survival-Associated Cancers

The differences of *PLOD2* gene expression were compared according to TNM stages of different tumors (data from TCGA database). Next, univariable and multivariable Cox regression analysis was used to compare the relationship between different *PLOD2* expression (divided into high and low expression groups with the median cutoff value) and prognosis. Prognosis includes OS (overall survival; period from the start of treatment to death from any cause), DSS (disease specific survival; cancer survival in the absence of other causes of death), and PFI (progression free interval; period from the start of treatment to disease progression or death from any cause). Subsequently, our findings were verified in the GSE84433 cohort.

### Procollagen-Lysine,2-Oxoglutarate 5-Dioxygenase 2 and Tumor Immunity

We used CIBERSORT(Newman et al., 2019) to explore the association of *PLOD2* gene expression with the level of immune infiltration in different types of cancer. CIBERSORT (https://CIBERSORT.stanford.edu/) is a tool for deconvolution of expression matrices of immune cell subtypes based on the principle of linear support vector regression, and can be used to estimate immune cell infiltration with data from RNA-Seq. Then we used xCell algorithm (Aran et al., 2017) and MCP-Counter algorithm (Becht et al., 2016) to verify the result of CIBERSORT.

In the tumor microenvironment, immune cells and stromal cells are two major non-tumor components (Bejarano et al., 2021; Dey et al., 2021). The immune score and stromal score calculated based on ESTIMATE algorithm (Yoshihara et al., 2013). The ESTIMATE algorithm produces three scores on the basis of single sample Gene Set Enrichment Analysis (ssGSEA): stromal score, immune score, and estimate score. In this study, we estimated these 3 scores and then calculated the relationship between these scores and *PLOD2* expression.

Furthermore, we examined the correlation between *PLOD2* expression and immune checkpoint-related genes (*BTLA*, *CD200*, *TNFRSF14*, *NRP1*, *LAIR1*, *TNFSF4*, *CD244*, *LAG3*, *ICOS*, *CD40LG*, *CTLA4*, *CD48*, *CD28*, *CD200R1*, *HAVCR2*, *ADORA2A*, *CD276*, *KIR3DL1*, *CD80*, *PDCD1*, *LGALS9*, *CD160*, *TNFSF14*, *IDO2*, *ICOSLG*, *TMIGD2*, *VTCN1*, *IDO1*, *PDCD1LG2*, *HHLA2*, *TNFSF18*, *BTNL2*, *CD70*, *TNFSF9*, *TNFRSF8*, *CD27*, *TNFRSF25*, *VSIR*, *TNFRSF4*, *CD40*, *TNFRSF18*, *TNFSF15*, *TIGIT*, *CD274*, *CD86*, *CD44*, *TNFRSF9*, shown in Supplementary Figure S4) using R software.

Next, we analyzed the relationship between *PLOD2* expression and TMB, MSI, and TNB. TMB is usually defined as the number of somatic nonsynonymous mutations or all mutations occurring per MB in the gene region detected by whole-exome sequencing or targeted sequencing in one tumor sample (Passaro et al., 2020). Somatic mutations calculated by TMB include point mutations and insertion/deletion mutations (Valero et al., 2021). TNB is an indicator of the total number of neoantigens in tumor cells, usually expressed as the number of tumor neoantigens per million bases of tumor genomic region (Wang et al., 2021). The combination of TMB and TNB can better predict the efficacy of immunotherapy. MSI, the insertion or loss of base pairs in microsatellite regions due to replication errors, was first identified in colorectal cancer and is thought to be a feature of hereditary non-polyposis colorectal cancer (Lynch syndrome) (Vilar and Gruber, 2010) and has since been found in a variety of sporadic tumors.

We downloaded the *PLOD2* genetic mutation data, transcriptome data, and clinical data from the TCGA database. To identify the somatic mutations of the patients with *PLOD2* in the TCGA database, mutation data were downloaded and visualized using the “maftools” package in R software. Horizontal histogram showed the genes have the higher mutation frequency in patients with *PLOD2*.

Finally, we evaluated the relationship between the expression of *PLOD2* and 5 DNA repair genes (MMRs: *MLH1*, *MSH2*, *MSH6*, *PMS2*, *EPCAM*) and 4 methyltransferases (*DNMT1*, *DNMT2*, *DNMT3A*, *DNMT3B*) genes.

### Gene Set Enrichment Analysis

Using JAVA (http://software.broadinstitute.org/gsea/index.jsp), we conducted GSEA to assess for possible underlying mechanisms based on the ‘Molecular Signatures Database’ of c5.all.v7.1.symbols and c2.cp.kegg.v7.1.symbols. When the number of random sample arrangements was 100 and the significance threshold was *p* < 0.05, R software and Bioconductor (http://bioconductor.org/) were applied to visualize our results.

### Immunohistochemical Staining

Tissue sections were prepared from the paraffin-embedded tissue samples. Then *PLOD2* immunostaining was performed according to the instructions (proteintech 21214-1-AP, China). Immunohistochemical scoring was performed by semi-quantitative analysis (20 pairs of gastric cancer and adjacent tissues). Two pathologists analyzed and scored the immunohistochemistry of gastric tissue. Each slice was randomly observed for 5 high-power visual fields, and scored according to the percentage of positive cells (0–5%, 6–25%, 26–50%, 51–75%, 76–100% were recorded as 0, 1, 2, 3, and 4 points respectively) and the intensity of staining (0, 1, 2, and 3 points respectively for non-staining, light, medium, and deep). The total score was the sum of staining intensity and percentage of positive cells. Next, we validated the expression of PLOD2 in STAD and normal tissues in the Human Protein Atlas (HPA) database (Uhlén et al., 2015).

### Statistical Methods

The Wilcoxon log-rank test was used to determine the presence or absence of a markedly increased sum of gene expression z-scores for tumor tissues, as compared to adjacent normal tissues. The difference in *PLOD2* expression between different tumor stages was compared using the Kruskal–Wallis H test. Survival was analyzed using the K-M curves, log-rank test, and Cox proportional hazards regression model. Spearman’s test was used for correlation analysis. R language (version 3.6.0; R Foundation) was used for all analyses. A two-sided *p* < 0.05 indicated a statistically significant difference.

## Results

### Pan-Cancer Expression Landscape of Procollagen-Lysine,2-Oxoglutarate 5-Dioxygenase 2

**FIGURE 1:**
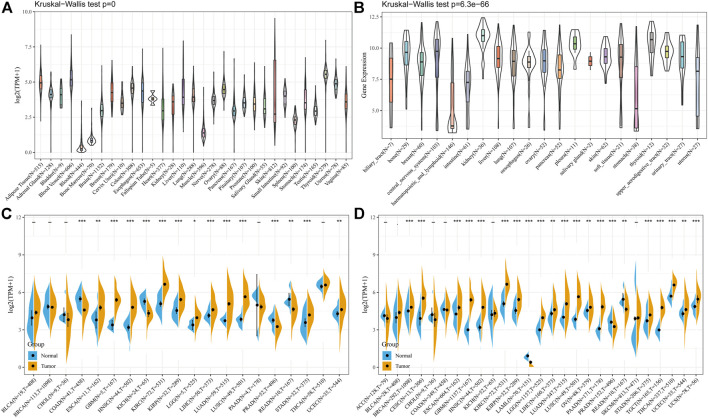
The *PLOD2* expression level in human pan-cancer analyses. **(A)** Expression of *PLOD2* in normal tissues in GTEx. **(B)** Expression of *PLOD2* in CCLE. **(C)** The level of *PLOD2* in TCGA. **(D)** The expression level in TCGA combined with GTEx. The blue and yellow bar graphs indicate normal and tumor tissues, respectively. *p < 0.05; **p < 0.01; ***p < 0.001. The significance of the two groups of samples passed the Wilcox test.

Firstly, we analyzed the expression levels of *PLOD2* in 7858 normal samples using the GTEx dataset. As shown in Figure 1A, the differences in *PLOD2* gene expression were significant (*p <* 0.001) in 31 tissues. Subsequently, we analyzed data downloaded from the CCLE database for each tumor cell line. There were significant differences in expression among the 21 tumor cell lines (Figure 1B), with the highest in renal tumors. Further, we obtained the differences in *PLOD2* from the TCGA database between cancer and para-cancer in individual tumor samples; and as shown in Figure 1C, *PLOD2* was highly expressed in 11 (ESCA, GBM, HNSC, KIRC, KIRP, LGG, LIHC, LUAD, LUSC, STAD, UCEC) of 20 different tumors, lowly expressed in 4 tumors (COAD, KICH, PRAD, READ). Finally, considering the small number of normal samples in TCGA database, we integrated data from GTEx and TCGA database to analyze the differences expression in multiple tumors (Supplementary Table S1). As shown in Figure 1D, *PLOD2* was highly expressed in 18 tumors (BRCA, CESC, ESCA, GBM, HNSC, KIRC, KIRP, LGG, LIHC, LUAD, LUSC, OV, PAAD, STAD, TGCT, THCA, UCEC, UCS) and lowly expressed in 3 tumors (LAML, PRAD, READ).

To assess the levels of gene expression for all tumor stages, we compared *PLOD2* expression in patients with different stages. As demonstrated in Supplementary Figure S1, *PLOD2* expression was upregulated at the advanced stages in BLCA, COAD, HNSC, KIRC, KIRP, LIHC, LUAD, and READ.

### Screening of Procollagen-Lysine,2-Oxoglutarate 5-Dioxygenase 2 Survival Associated Cancers

In the OS analysis, Cox regression identified that high *PLOD2* expression was a risk factor for CESC (*p <* 0.001), CHOL (*p =* 0.032), HNSC (*p =* 0.005), KICH (*p <* 0.001), KIRC (*p* = 0.001), KIRP (*p* = 0.034), LGG (*p* < 0.001), LIHC (*p* < 0.001), LUAD (*p* = 0.001), MESO (*p* = 0.020), PAAD (*p* = 0.006), SARC (*p* = 0.005), and STAD (*p* = 0.001); however, it appeared to be a protective factor in LAML (*p* = 0.024) and SKCM (*p* = 0.049), as shown in Supplementary Figure S2A. The Cox regression analysis of DSS indicated that high *PLOD2* expression is a risk factor in CESC (*p* < 0.001), CHOL (*p* = 0.038), ESCA (*p* = 0.045), HNSC (*p* = 0.020), KICH (*p* < 0.001), KIRC (*p* < 0.001), LGG (*p* < 0.001), LIHC (*p* < 0.001), LUAD (*p* = 0.001), MESO (*p* = 0.007), PAAD (*p* = 0.004), SARC (*p* = 0.005) and STAD (*p* = 0.006) as illustrated in Supplementary Figure S2B. The Cox regression analysis of PFI revealed that higher *PLOD2* expression is a risk factor in CESC(*p* < 0.001), ESCA (*p* = 0.049), KICH (*p* < 0.001), KIRC (*p* < 0.001), LGG (*p* < 0.001), LIHC (*p* = 0.001), LUAD (*p* = 0.004), MESO (*p* = 0.028), PAAD (*p* = 0.015), SARC (*p* < 0.001) and STAD (*p* = 0.047). In the DFI analysis, Cox regression identified that high *PLOD2* expression was a risk factor for CESC (*p* = 0.010), CHOL (*p* = 0.038), KIRC (*p* = 0.023), LIHC (*p* = 0.019), LUAD (*p* = 0.022), PAAD (*p* < 0.001) and SARC (*p* = 0.007).

**FIGURE 2:**
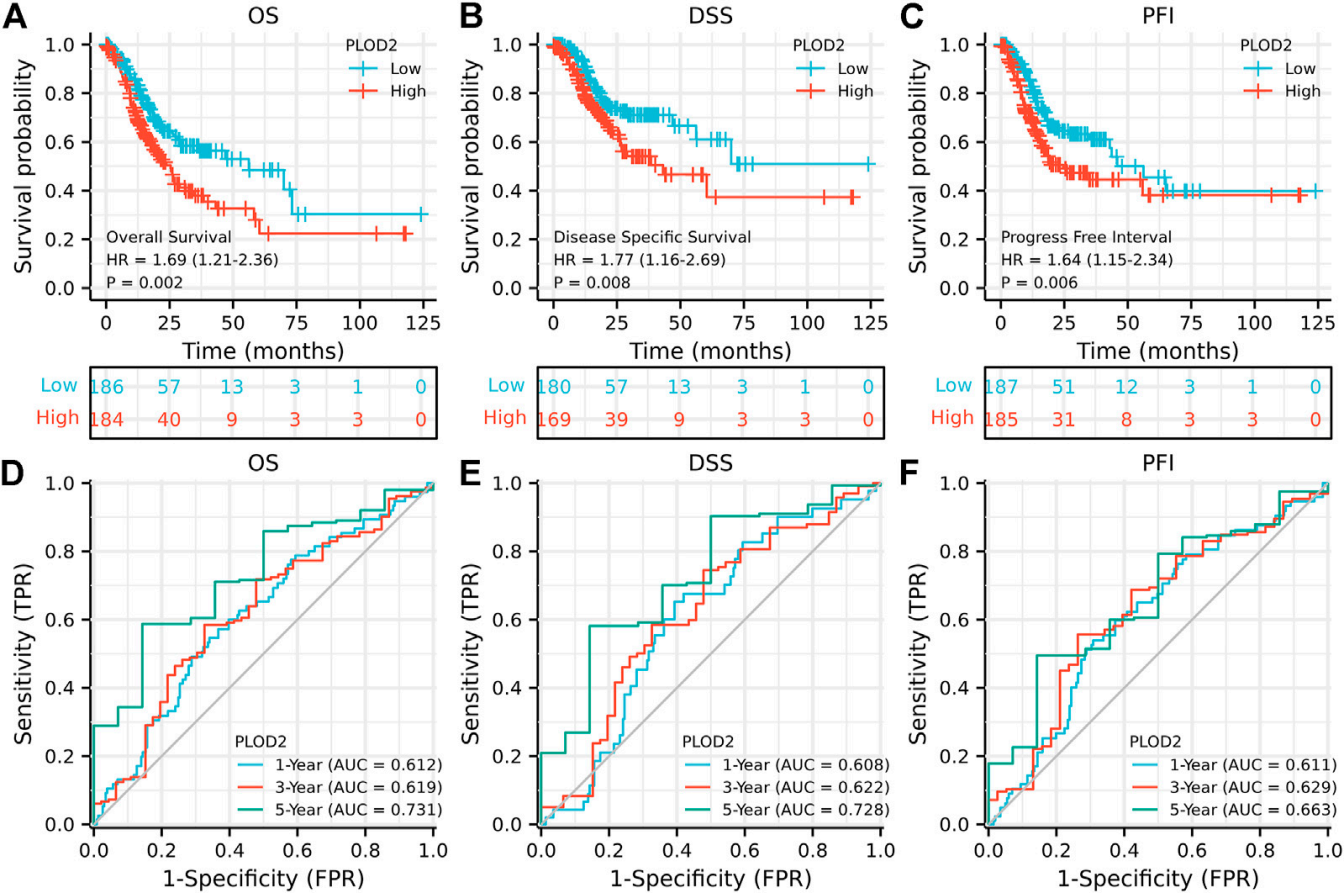
Prognostic analysis of *PLOD2* gene signature in STAD in the TCGA set. **(A)** overall survival **(B)** disease-specific survival **(C)** progression-free interval **(D)** ROC of overall survival **(E)** ROC of disease-specific survival **(F)** ROC of progression-free interval.

**FIGURE 3:**
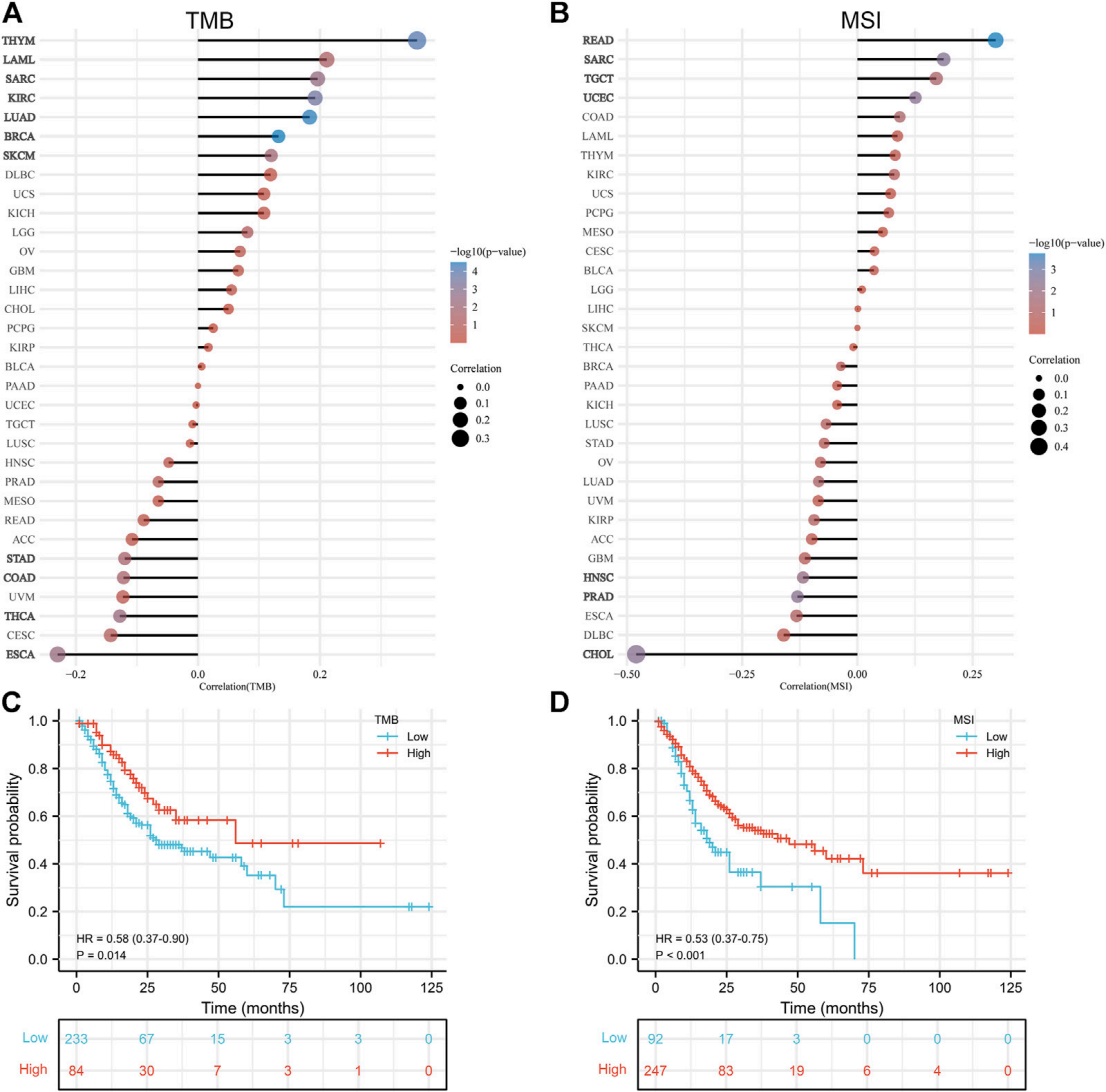
Correlation analysis of *PLOD2* expression with tumor mutational burden and microsatellite instability. **(A)** with TMB **(B)** with MSI **(C)** relationship between TMB and overall survival **(D)** relationship between MSI and overall survival.

We further analyzed the relationship between *PLOD2* gene expression and prognosis in gastric cancer patients in detail (Figure 2). Figures 2A–C showed the relationship between *PLOD2* gene expression and OS, DSS and PFI, respectively. In the OS analysis, as illustrated in Figure 2A, we split cases into high-risk and low-risk groups according to the median expression. The median OS time was significantly higher in the *PLOD2* low expression group than in the high expression group [HR 1.69 (95% CI 1.21–2.36), *p =* 0.002]. Time-dependent receiver operating characteristic (ROC) curves were plotted and the area under curve (AUC) values of the classifier to predict 1-, 3-, and 5-year OS were 0.612, 0.619, and 0.731, respectively (Figure 2D). In DSS analysis (Figure 2B) and PFI analysis (Figure 2C), hazard ratio was 1.77 (95% CI 1.16–2.69, *p* = 0.008) and 1.64 (95% CI 1.15–2.34, *p* = 0.006), respectively. AUC values of the classifier to predict 1-, 3-, and 5-year DSS were 0.608, 0.622, and 0.728, respectively (Figure 2E). AUC values of the classifier to predict 1-, 3-, and 5-year PFI were 0.611, 0.629, and 0.663, respectively (Figure 2F). As shown in Figure 3A, in the GSE84433 validation cohort (355 patients remained after deleting 2 patients who survived less than 1 month), the overall survival time of the PLOD2 low expression group was also significantly longer than that of the high expression group [HR 0.73, 95% CI (0.54–0.99), *p* = 0.041].

As shown in Table 1, univariate analysis showed that age (*p* = 0.005), T stage, N stage, M stage, pathologic stage and PLOD2 expression were significantly correlated with OS (all *p* < 0.05). However, in multivariate analysis, only age [HR: 1.731 (95% CI 1.194–2.508), *p* = 0.004], M stage [HR: 2.038 (95% CI 1.094–3.799), *p* = 0.025] and PLOD2 expression [HR: 1.484 (95% CI 1.034–2.131), *p* = 0.032] were significantly correlated with prognosis. In the GSE84433 validation cohort, both univariate [HR 1.369 95% CI (1.013–1.851), *p* = 0.041] and multivariate [HR 1.434 95% CI (1.057–1.947), *p* = 0.021] analysis, the overall survival of PLOD2 high expression group was significantly worse than that of low expression group (Figure 3B).

**TABLE 1:** Univariate and multivariate Cox regression analysis of overall survival.

Characteristics	Total(N)	Univariate analysis		Multivariate analysis	
		Hazard ratio (95% CI)	*p* value	Hazard ratio (95% CI)	*p* value
Age	367				
≤65	163	References			
>65	204	1.620 (1.154–2.276)	**0.005**	1.731 (1.194–2.508)	**0.004**
Gender	370				
Male	237	References			
Female	133	0.789 (0.554–1.123)	0.188		
T stage	362				
T1&T2	96	References			
T3&T4	266	1.719 (1.131–2.612)	**0.011**	1.252 (0.741–2.118)	0.401
N stage	352				
N0	107	References			
N1	97	1.629 (1.001–2.649)	**0.049**	1.300 (0.712–2.374)	0.394
N2	74	1.655 (0.979–2.797)	0.060	1.359 (0.647–2.853)	0.418
N3	74	2.709 (1.669–4.396)	**<0.001**	2.032 (0.952–4.339)	0.067
M stage	352				
M0	327	References			
M1	25	2.254 (1.295–3.924)	**0.004**	2.038 (1.094–3.799)	**0.025**
Pathologic stage	347				
Stage I and II	160	References			
Stage III and IV	187	1.947 (1.358–2.793)	**<0.001**	1.144 (0.600–2.181)	0.683
PLOD2	370				
Low	186	References			
High	184	1.693 (1.215–2.360)	**0.002**	1.484 (1.034–2.131)	**0.032**

The bold values means P<0.05.

### Procollagen-Lysine,2-Oxoglutarate 5-Dioxygenase 2 Level and Immune Infiltration

**FIGURE 4:**
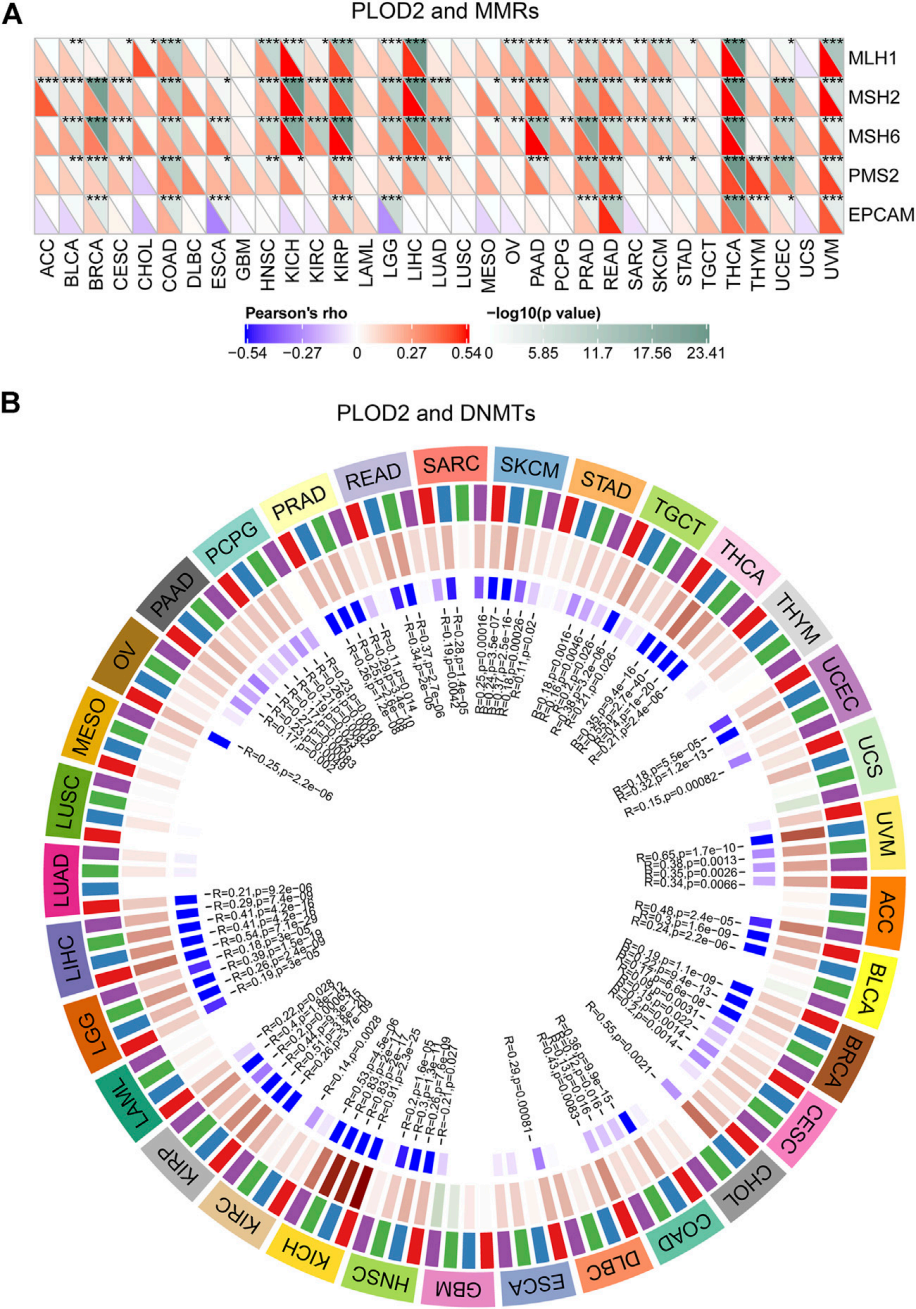
Relationship between *PLOD2* expression and MMRS and methyltransferase in pan-cancer. **(A)** Relationship between *PLOD2* expression and mutation of 5 MMRs genes. **(B)** Relationship between 4 methyltransferases and *PLOD2* expression. Red, blue, green, and purple colors are indicated *DNMT1*, *DNMT2, DNMT3A*, and *DNMT3B*, respectively.

Tumor-infiltrating lymphocytes are independent predictors of lymph node status and survival in cancer precursors. Immune infiltrating cells were analyzed using CIBERSORT. Although the tumor types differed, the relationship between *PLOD2* expression and immune cells was similar. For example, *PLOD2* expression was significantly negatively correlated with memory B cell, activated NK cell, Plasma cell, CD8 T cell, follicular helper T cell, regulatory T cell in most tumors; and significantly positively correlated with Macrophages M0, Macrophages M1, Macrophages M2, activated Mast cell, resting NK cell, CD4 memory activated T cell, CD4 memory resting T cell. As shown in Figure 4C, PLOD2 was significantly correlated with 19 immune checkpoints in patients with STAD, of which 80% (14/19, CD200, CD276, CD28, CD44, CD80, CD86, HAVCR2, LAIR1, NRP1, PDCD1LG2, TNFRSF25, TNFRSF9, TNFSF14, TNFSF18, TNFSF4) was positively correlated.

Next, we analyzed the relationship between *PLOD2* expression and infiltrating immune cells in gastric cancer based on the xCELL algorithm. As shown in Figure 4B, the proportion of T cell CD4^+^ Th1, Macrophage, Macrophage M1, Plasmacytoid dendritic cell, B cell, Monocyte, Neutrophil, and Endothelial cell were significantly higher in the *PLOD2* high expression group than low expression group. Contrarily, the proportion of B cell plasma, microenvironment score, T cell CD8^+^ effector memory, T cell CD8^+^ central memory, Class-switched memory B cell, B cell memory, Granulocyte-monocyte progenitor, Hematopoietic stem cell and stroma score were higher in the *PLOD2* low expression group. we also used MCP-Counter deconvolution methods to verify our results (Supplementary Figures S7A,B). In the GSE84433 validation cohort, macrophage M0 (*p* < 0.05) and macrophage M2 (*p* < 0.05) increased significantly in the PLOD2 high expression group, while T cell CD4 + memory activated (*p* < 0.001) decreased significantly (Figure 3C).

Numerous studies indicated that the tumor immune microenvironment has an important role in tumor development. As shown in Table 2, in 9 kinds of tumors (BLCA, COAD, GBM, KIRC, LGG, OV, PAAD, PCPG, and PRAD), immune scores were positively correlated with the expression of *PLOD2* and negatively correlated with the expression of CESC, SARC, TGCT, THCA, THYM, and UCEC. For stromal scores, 22 kinds of tumors (BLCA, BRCA, COAD, DLBC, ESCA, GBM, HNSC, KIRC, LAML, LGG, LUAD, LUSC, OV, PAAD, PCPG, PRAD, READ, SARC, SKCM, STAD, TGCT and THYM) were positively correlated with the expression of *PLOD2*. Combining immune scores and stromal scores gives estimate scores. *PLOD2* gene expression was positively correlated with estimate scores in 17 tumors (BLCA, BRCA, COAD, DLBC, ESCA, GBM, HNSC, KIRC, LAML, LGG, LUAD, OV, PAAD, PCPG, PRAD, READ, and STAD, in Supplementary Figure S3).

**TABLE 2:** Correlation analysis of *PLOD2* expression with immune scores, stromal scores, and estimate scores.

Cancer type	Immune scores		Stromal scores		Estimate scores	
	R	P	R	P	R	P
ACC	0.056	0.624	0.103	0.366	0.080	0.484
BLCA	**0.221**	<0.001	**0.266**	<0.001	**0.260**	<0.001
BRCA	0.038	0.203	**0.192**	<0.001	**0.108**	<0.001
CESC	−**0.168**	0.003	−0.056	0.332	−**0.126**	0.028
CHOL	0.042	0.806	0.255	0.133	0.089	0.605
COAD	**0.117**	0.012	**0.327**	<0.001	**0.243**	<0.001
DLBC	−0.145	0.326	**0.606**	<0.001	**0.328**	0.024
ESCA	0.044	0.579	**0.281**	<0.001	**0.180**	0.022
GBM	**0.192**	0.013	**0.310**	<0.001	**0.263**	<0.001
HNSC	0.014	0.751	**0.227**	<0.001	**0.132**	0.003
KICH	−0.103	0.415	0.095	0.449	−0.015	0.908
KIRC	**0.111**	0.010	**0.283**	<0.001	**0.204**	<0.001
KIRP	−0.089	0.131	0.058	0.329	−0.018	0.763
LAML	0.035	0.672	**0.309**	<0.001	**0.179**	0.028
LGG	**0.476**	<0.001	**0.512**	<0.001	**0.505**	<0.001
LIHC	0.023	0.663	−0.055	0.293	−0.017	0.739
LUAD	0.030	0.500	**0.142**	0.001	**0.095**	0.031
LUSC	−0.033	0.462	**0.100**	0.025	0.030	0.498
MESO	0.016	0.883	0.209	0.054	0.095	0.382
OV	**0.173**	<0.001	**0.307**	<0.001	**0.257**	<0.001
PAAD	**0.228**	0.002	**0.501**	<0.001	**0.369**	<0.001
PCPG	**0.159**	0.032	**0.331**	<0.001	**0.252**	<0.001
PRAD	**0.089**	0.049	**0.227**	<0.001	**0.163**	<0.001
READ	0.068	0.384	**0.371**	<0.001	**0.245**	0.001
SARC	−**0.198**	0.001	−**0.181**	0.004	−**0.202**	0.001
SKCM	0.006	0.904	**0.159**	<0.001	0.076	0.097
STAD	0.053	0.307	**0.416**	<0.001	**0.252**	<0.001
TGCT	−**0.326**	<0.001	**0.189**	0.018	−0.141	0.079
THCA	−**0.262**	<0.001	−**0.152**	<0.001	−**0.233**	<0.001
THYM	−**0.316**	<0.001	**0.192**	0.037	−0.053	0.565
UCEC	**0.234**	<0.001	−**0.145**	<0.001	−**0.211**	<0.001
UCS	−0.154	0.255	−0.006	0.963	−0.107	0.431
UVM	0.140	0.216	−0.166	0.141	−0.151	0.180

The correlation coefficient with *p* value less than 0.05 is expressed in bold.

Finally, we collected more than forty common immune checkpoint genes to analyze the relationship between *PLOD2* gene expression and immune checkpoint gene expression (Supplementary Figure S4). Among them, *TNFRSF14*, *NRP1*, *LAIR1*, *TNFSF4*, *CD276*, *CD80*, *PDCD1LG2*, *CD274*, *CD86*, and *CD44* were significantly positively correlated with *PLOD2* expression.

### Correlation Analysis With TMB and MSI

**FIGURE 5:**
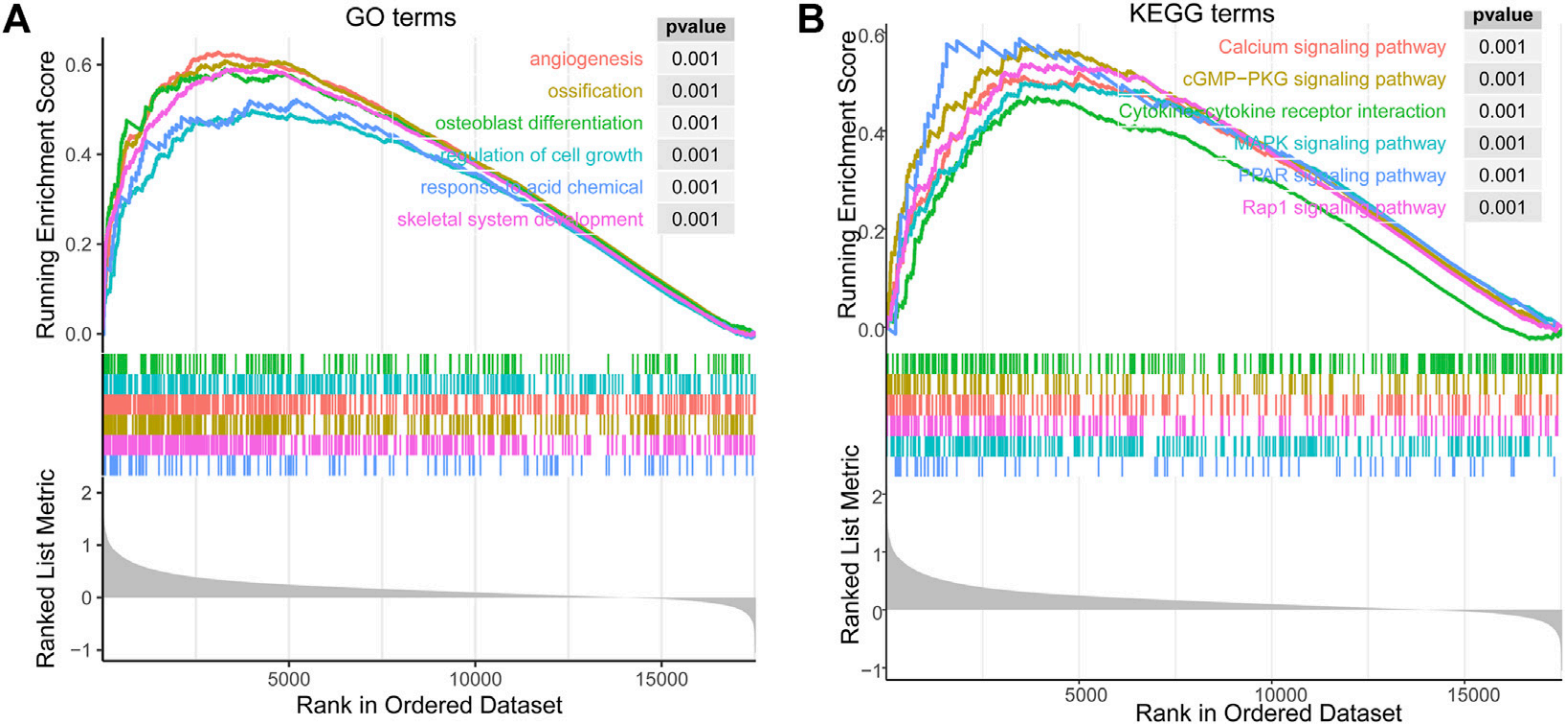
The enrichment results of GSEA correlated with *PLOD2* expression in STAD. **(A)** top 6 significant enrichment GO terms. **(B)** top 6 KEGG terms.

In general, high TMB is associated with better OS; higher TMB is associated with better response to immune checkpoint inhibition. The association between TMB and *PLOD2* expression was evaluated, as seen in Figure 5A. *PLOD2* expression was positively correlated with TMB in BRCA (*p <* 0.001), LUAD (*p <*0.001), THYM (*p* < 0.001), KIRC (*p <* 0.001), SARC (*p =* 0.003), SKCM (*p =* 0.009), and LAML (*p =* 0.041); but negatively correlated with ESCA (*p =* 0.004), THCA (*p =* 0.005), STAD (*p =* 0.021) and COAD (*p =* 0.022). In addition, in the low expression group of PLOD2, there was a significant negative correlation between PLOD2 and TMB (r = -0.160, *p* = 0.030), but there was no significant correlation in the high expression group (r = 0.013, *p* = 0.862). As shown in Figure 5C, TMB was significantly correlated with OS. OS in the TMB high group was significantly longer than that in the low group [HR 0.58 95% CI (0.37–0.90), *p* = 0.014].

*PLOD2* was positively correlated with MSI in READ (*p <* 0.001), SARC (*p =* 0.003), UCEC (*p =* 0.003), and TGCT (*p =* 0.048); but negatively correlated with CHOL (*p =* 0.003), PRAD (*p =* 0.004) and HNSC (*p =* 0.008), as shown in Figure 5B. With similarly, the results of TMB, PLOD2 was negatively correlated with MSI in the low expression group (r = -0.148, *p* = 0.043), but not in the high expression group (r = -0.049, *p* = 0.505). The OS of high MSI group was significantly longer than that of low group, in Figure 5D [HR 0.53 95% CI (0.37–0.75), *p* < 0.001].

### Relationship Between Procollagen-Lysine,2-Oxoglutarate 5-Dioxygenase 2 Somatic Mutation, Mismatch Repairs and DNA Methyltransferase

We downloaded mutect-processed mutation data from TCGA to analyze the mutation of *PLOD2* gene in these tumors. As Supplementary Figure S6A demonstrated, the proportion of *PLOD2* mutations in each tumor, which ranged from 5.09% (UCEC) to 0.23% (OV). Supplementary Figure S6B shown the distribution of mutations in the top 3 tumors, UCEC (5.09%), COAD (2.76%) and STAD (2.75%). In STAD, the most common of the was Missense Mutation, followed by Frame Shift Del and Nonsense Mutation.

**FIGURE 6:**
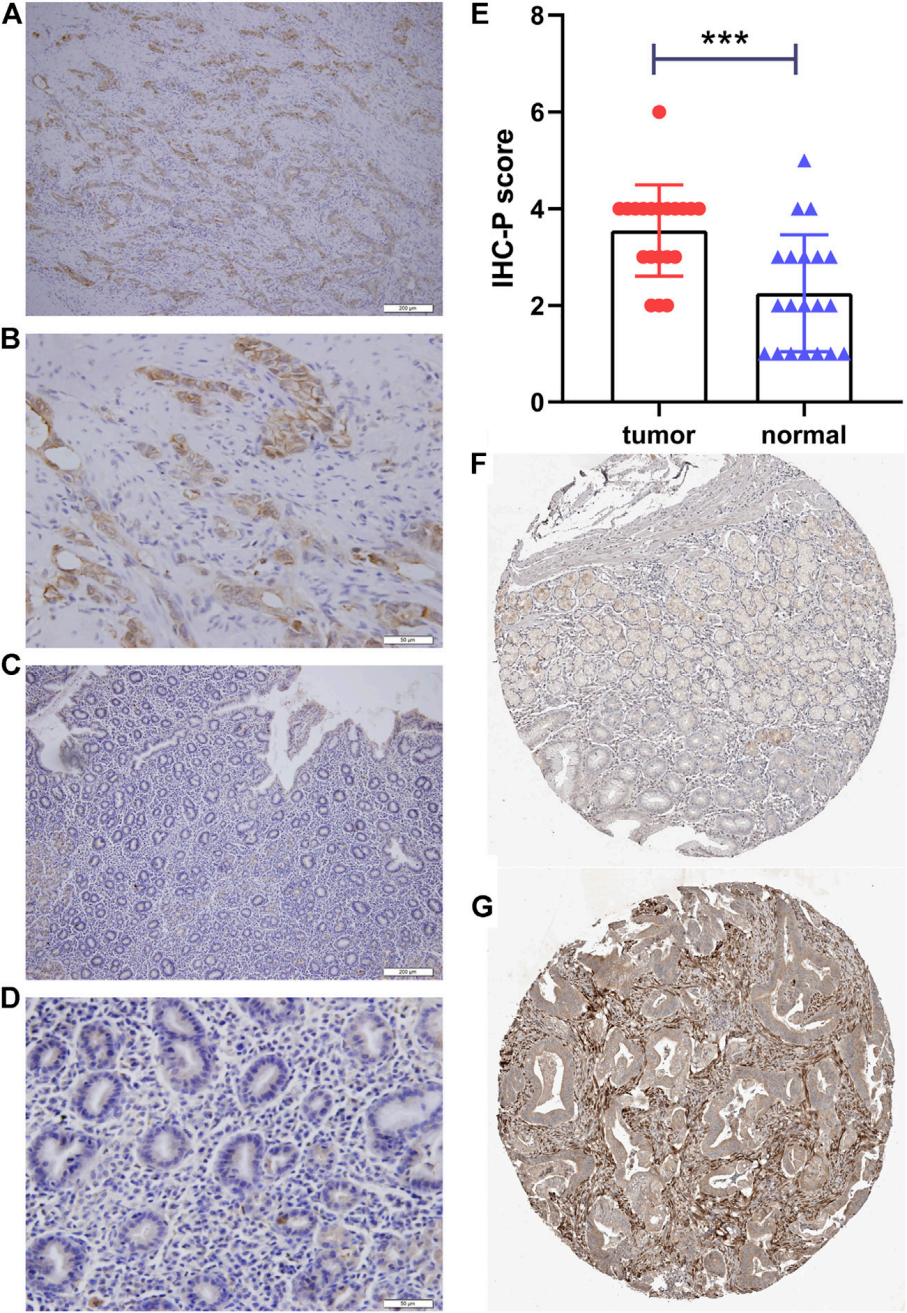
Immunohistochemical staining of *PLOD2*. **(A)** gastric cancer, ×10. **(B)** gastric cancer, ×40. **(C)** adjacent tissue, ×10. **(D)** adjacent tissue, ×40. **(E)** Statistical analysis of cancer and adjacent tissue. **(F)** validation the expression of PLOD2 in normal tissues (Patient ID 1650, staining low) in the Human Protein Atlas (HPA) database **(G)** validation the expression of PLOD2 in STAD (Patient ID 2557, staining medium) in the HPA database ***p < 0.001.

MMRs (Mismatch Repairs) was the repair of nucleotide sequences to normal in DNA molecules containing mismatched bases. Thus, the MMR system was a safety and security system *in vivo* that maintains the integrity and stability of genetic material. As shown in Figure 6A, the expression of *PLOD2* was positively correlated with *MLH1*, *MSH2*, and *MSH6* in a variety of tumors. In STAD, *PLOD2* expression was positively correlated with *MLH1*, *MLH6*, *and PSM2*.

In addition, a close relationship was observed between *PLOD2* expression and mutations in 4 methyltransferases (*DNMT1*, *DNMT2*, *DNMT3A*, *DNMT3B*) in several cancer types (Figure 6B). For example, in STAD, POLD2 expression was positively correlated with *DNMT3A* (marked green, R = 0.18, *p* = 0.002) and *DNMT3B* (marked purple, R = 0.16, *p =* 0.005) expression.

### Functional Analysis

**FIGURE 7:**
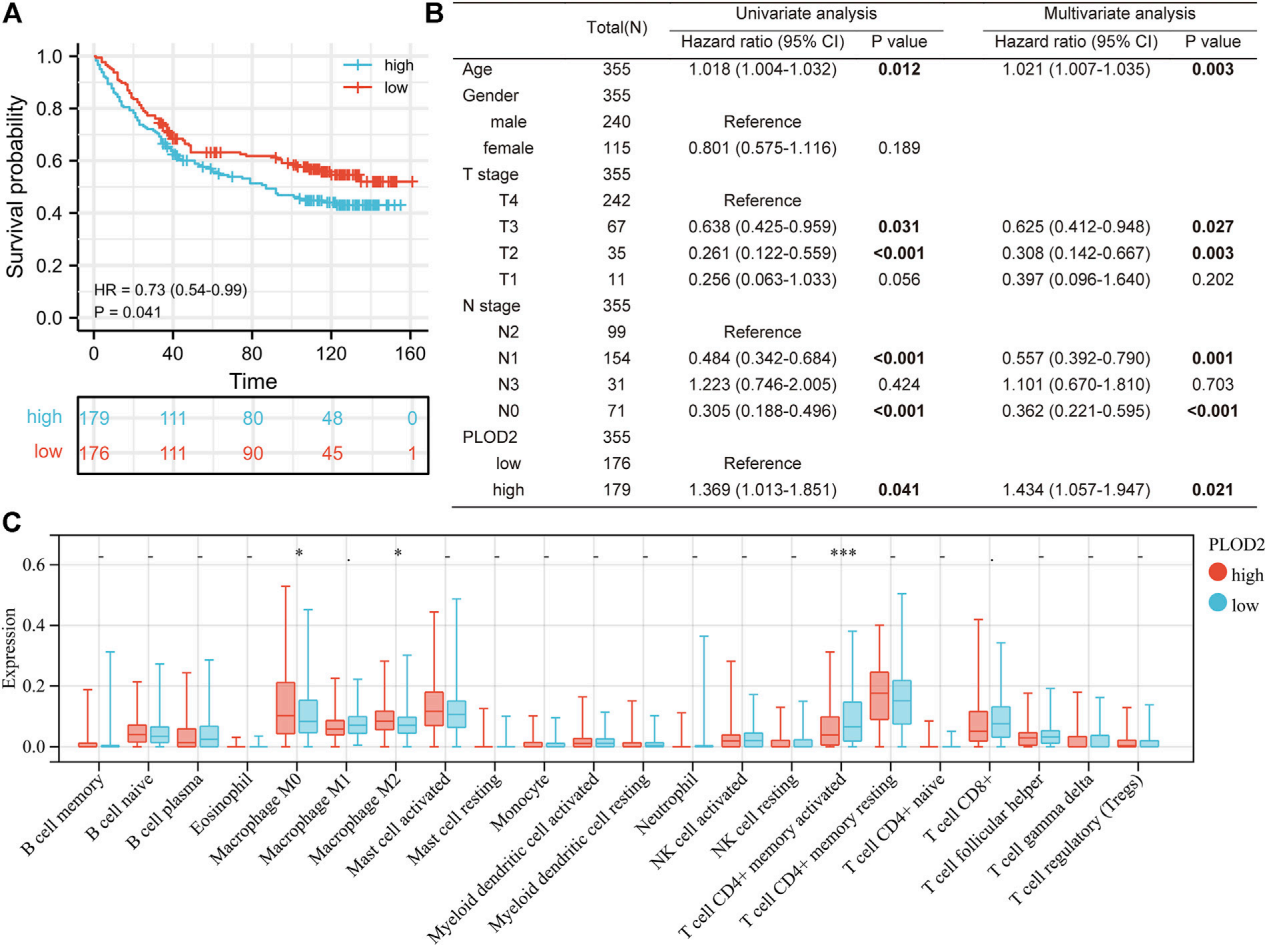
Validation PLOD2 expression in GSE84433 cohort. **(A)** survival curve of high and low PLOD2 expression. **(B)** univariate and multivariate analysis of overall survival in GSE84433 cohort. **(C)** immune infiltration difference between high and low PLOD2 expression in GSE84433 cohort, based on CIBERSORT deconvolution method.

The biological effect of *PLOD2* expression was assessed using GSEA. In STAD, Figure 7A showed top 6 significant enrichment GO terms: angiogenesis (GO:0001525); ossification (GO:0001503); osteoblast differentiation (GO:0001649); regulation of cell growth (GO:0001558); response to acid chemical (GO:0001101); skeletal system development (GO:0001501). The top 6 KEGG terms also showed significant enrichment (Figure 7B): Calcium signaling pathway (hsa04020); cGMP−PKG signaling pathway (hsa04022); Cytokine−cytokine receptor interaction (hsa04060); MAPK signaling pathway (hsa04010); PPAR signaling pathway (hsa03320); Rap1 signaling pathway (hsa04015).

### Immunohistochemical Staining

**FIGURE 8:**
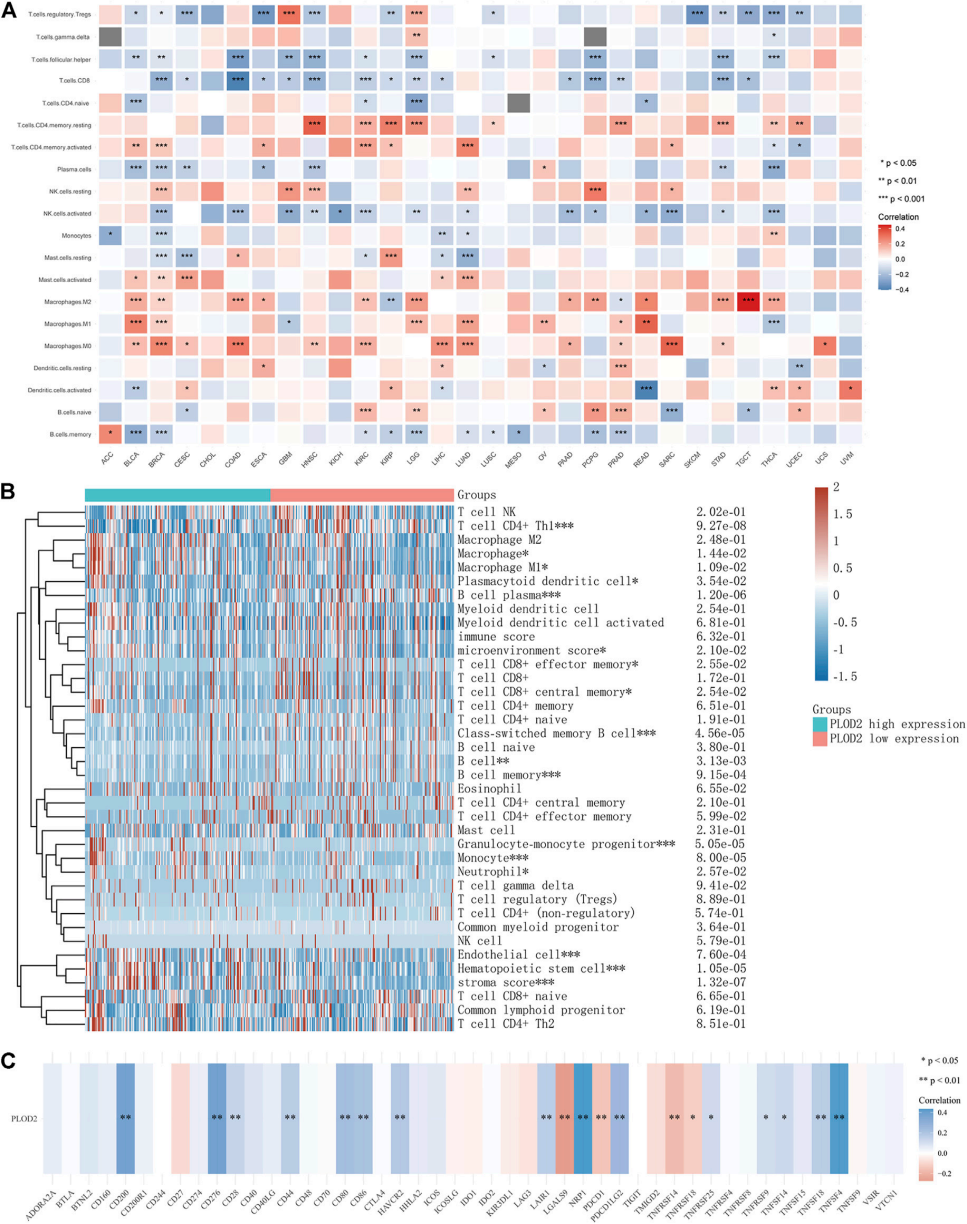
Relationship between *PLOD2* expression and immune cells in pan-cancer. **(A)** CIBERSORT predicts that *PLOD2* expression is correlated with immunocytes. **(B)** Heatmap of *PLOD2* expression and infiltrating immune cells in STAD based on the xCELL algorithm. **(C)** relationship between PLOD2 and immune checkpoint in STAD.

As shown in Table 3, the average age of the 20 gastric cancer patients was 62 years old, and women accounted for 35%. Among the clinical stages, stage III or IV accounted for 55%, signet ring cell carcinoma accounted for 25%, and diffuse, intestinal and mixed accounted for 55%, 15%, and 30% respectively. We note that clinical stage III or IV in PLOD2-high group accounted for 75%, and Lauren’s type was mainly diffuse and mixed; while clinical stage III or IV in PLOD2-high group accounted for 25%. Lauren’s classification was mainly diffuse and intestinal. However, due to the small sample size, there was no significant difference. Figure 8 showed the immunohistochemical staining results of 20 pairs of gastric cancer and corresponding adjacent tissues. In the gastric cancer group, *PLOD2* was significantly overexpressed, while the expression in adjacent tissues was low. The median values of the two groups were 4 and 2 respectively. There was significant difference in staining score (*p <* 0.001, Figure 8E). Figures 8A,B were cancer tissues, and Figures 8C,D were adjacent tissues. The results of HPA database also showed that the expression of PLOD2 in STAD was higher than that normal gastric tissue (Figures 8F,G).

**TABLE 3:** Relationship between PLOD2 expression and clinicopathology.

Characteristic	Total	PLOD2-high	PLOD2-low	*p*
*n*	20	12	8	
age, mean ± SD	62 ± 13	61 ± 11	64 ± 16	0.594
gender, *n* (%)				>0.999
female	7 (35%)	4 (33.3%)	3 (37.5%)	
male	13 (65%)	8 (66.7%)	5 (62.5%)	
stageT, *n* (%)				0.170
1 or 2	10 (50.0%)	4 (33.3%)	6 (75%)	
3 or 4	10 (50.0%)	8 (66.7%)	2 (25%)	
stageN, *n* (%)				0.289
0	3 (15%)	3 (25%)	0 (0%)	
1	1 (5%)	0 (0%)	1 (12.5%)	
2	8 (40%)	4 (33.3%)	4 (50%)	
3	8 (40%)	5 (41.7%)	3 (37.5%)	
stageM, *n* (%)				>0.999
0	18 (90%)	11 (91.7%)	7 (87.5%)	
1	2 (10%)	1 (8.3%)	1 (12.5%)	
clinical stage, *n* (%)				0.065
1 or 2	9 (45%)	3 (25%)	6 (75%)	
3 or 4	11 (55%)	9 (75%)	2 (25%)	
differentiation grade, *n* (%)				0.465
poorly	11 (55%)	5 (41.7%)	6 (75%)	
moderately	8 (40%)	6 (50%)	2 (25%)	
well	1 (5%)	1 (8.3%)	0 (0%)	
signet-ring, *n* (%)				0.603
non–signet-ring	15 (75%)	8 (66.7%)	7 (87.5%)	
signet-ring	5 (25%)	4 (33.3%)	1 (12.5%)	
Lauren type, *n* (%)				0.052
diffuse	11 (55%)	5 (41.7%)	6 (75%)	
intestinal	3 (15%)	1 (8.3%)	2 (25%)	
mixed	6 (30%)	6 (50%)	0 (0%)	
HER-2, *n* (%)				>0.999
0 or 1+	12 (60%)	7 (58.3%)	5 (62.5%)	
2+ or 3+	8 (40%)	5 (41.7%)	3 (37.5%)	

## Discussion

The present study aimed to demonstrate a comprehensive workflow for pan-cancer analysis and to extensively investigate the role of *PLOD2* as it related to various cancers. Based on our results, we found that *PLOD2* overexpression was associated with prognosis in a variety of tumors (CHOL, HNSC, KIRC, KIRP, LAML, LUAD, MESO, PAAD, SARC, SKCM, and STAD) based on Cox proportional risk models and KM survival analysis. We focused on the relationship between PLOD2 expression and STAD. Univariate and multivariate analysis showed that the overall survival time of PLOD2 high expression group was significantly less than low group. Immunohistochemical results also showed that the PLOD2 expression in gastric cancer was significantly higher than normal tissues.

In order to investigate the research status of PLOD2 and gastric cancer, we conducted a search on the PubMed using the following search strategy: [“stomach neoplasms” (Title/Abstract) OR “stomach neoplasms” (MeSH Terms) OR “gastric adenocarcinoma” (Title/Abstract)] AND [“PLOD2” (Title/Abstract)]. Finally, seven studies were found (Kiyozumi et al., 2018; Li S. S et al., 2020; Luo et al., 2020; Wang et al., 2020; Dai et al., 2021; Li et al., 2021; Song et al., 2021). Dai et al. constructed a prognostic model for five genes including *PLOD2*, which was subsequently validated by RT-PCR in normal tissue and gastric cancer cell lines (Dai et al., 2021). However, their study did not perform analyses related to tumor immunity (including infiltrating immune cells, TMB, etc.). Similarly, Li J et al.(Li et al., 2021), Li SS et al. (Li S. S et al., 2020), Luo et al. (Luo et al., 2020), and Song et al.(Song et al., 2021) were also constructed multiple genes (including *PLOD2*) prognostic model, but all lacked tumor immune-related analysis or only had comparisons of different immune cell classifications. Kiyozumi et al. study showed that *PLOD2* promotes cell invasion and migration in gastric cancer under hypoxic conditions and leads to dissemination to the peritoneum, *in vitro* (Kiyozumi et al., 2018). This might be even better when coupled with a *PLOD2* knockout or overexpression mouse model. Wang et al. conducted a study on the relationship between *PLOD2* gene expression and gastric cancer chemotherapy (Wang et al., 2020). Their results showed that *PLOD2* knockdown in BGC823 cells significantly reduced the IC_50_ value of 5-FU, which contributed to the reduction of migration and invasion and promoted apoptosis of gastric cancer cells. The opposite result appeared in *PLOD2* overexpressing MGC803 cells. *In vivo* experiments showed that knockdown of *PLOD2* gene enhanced the inhibitory effect of 5-FU on the growth of transplanted tumors in nude mice. It is particularly unfortunate that the study was only cellular and animal-based, and extrapolation to human gastric cancer requires further validation. In brief, all of the above studies have their own merits and there were many areas for further improvement also.

Our study showed that both OS, DSS, and PFI suggested that the prognosis of PLOD2 high group of was significantly worse than that low group. This may be related to the following reasons. Firstly, the immune cell infiltration in the low group was more abundant (DC, M1 macrophages, CD4 + T cells, CD8 + T cells higher than PLOD2 low group (Figure 4B). Secondly, immune checkpoint gene was also significantly overexpressed in the high expression group. Thirdly, the tumor stroma score in the high group was significantly higher than low group (shown in Figure 4B). This indicates that the proportion of non-immune cells [e.g., cancer associated fibroblasts (Chen and Song, 2019)] was aplenty in the high group. Derks et al. showed that the infiltration of non-immune cells (such as fibroblasts and stromal cells) was associated with poor prognosis in gastroesophageal adenocarcinomas (Derks et al., 2020). In addition, immunohistochemical results showed that in the high PLOD2 group, the clinical stages were mainly stage III and stage IV, and the proportion of signet ring cell carcinoma was also higher. Signet ring cell carcinomas was usually “cold tumor” (i.e., lack of immune infiltration) (Garcia-Pelaez et al., 2021; Monster et al., 2022). Therefore, we speculated that the high expression of PLOD2 and poor prognosis may be related to immune infiltration and pathological types. However, further animal experiments were needed to prove it.

In conclusion, we have found that *PLOD2* can serve as a valuable prognostic biomarker for some tumors, especially gastric cancer. We believe that these findings may lay the groundwork for prospective functional experiments and eventually have an impact in clinical work.

## Data Availability

The datasets presented in this study can be found in online repositories. The names of the repository/repositories and accession number(s) can be found in the article/Supplementary Material.

## Abbreviations

CCLE, cancer cell line encyclopedia; TCGA, the cancer genome atlas; PLOD2, procollagen-lysine,2-oxoglutarate 5-dioxygenase 2; ECM, extracellular matrix; TMB, tumor mutational burden; MSI, microsatellite instability; TNB, tumor neoantigen burden; GSEA, gene set enrichment analysis; GTEx, genotype-tissue expression; OS, overall survival; DFI, disease free interval; DSS, disease specific survival; PFI, progression free interval; KM, Kaplan-Meier.
