# Supplementary material for: Pan-Cancer Analyses Reveal Oncogenic and Immunological Role of PLOD2
Source: Front Genet. 2022 May 2;13:864655. doi: 10.3389/fgene.2022.864655 (PMC9108334; doi:10.3389/fgene.2022.864655)
Supplement: Supplementary file 1 [file DataSheet1.PDF]

Table S1. 33 types of human cancers employed in our research

Cancer type	Full name
ACC	Adrenocortical carcinoma
BLCA	Bladder Urothelial Carcinoma
BRCA	Breast invasive carcinoma
CESC	Cervical squamous cell carcinoma and endocervical adenocarcinoma
CHOL	Cholangiocarcinoma
COAD	Colon adenocarcinoma
DLBC	Lymphoid Neoplasm Diffuse Large B-cell Lymphoma
ESCA	Esophageal carcinoma
GBM	Glioblastoma multiforme
HNSC	Head and Neck squamous cell carcinoma
KICH	Kidney Chromophobe
KIRC	Kidney renal clear cell carcinoma
KIRP	Kidney renal papillary cell carcinoma
LAML	Acute Myeloid Leukemia
LGG	Brain Lower Grade Glioma
LIHC	Liver hepatocellular carcinoma
LUAD	Lung adenocarcinoma
LUSC	Lung squamous cell carcinoma
MESO	Mesothelioma
OV	Ovarian serous cystadenocarcinoma
PAAD	Pancreatic adenocarcinoma
PCPG	Pheochromocytoma and Paraganglioma
PRAD	Prostate adenocarcinoma
READ	Rectum adenocarcinoma
SARC	Sarcoma
SKCM	Skin Cutaneous Melanoma
STAD	Stomach adenocarcinoma
TGCT	Testicular Germ Cell Tumors
THCA	Thyroid carcinoma
THYM	Thymoma
UCEC	Uterine Corpus Endometrial Carcinoma
UCS	Uterine Carcinosarcoma
UVM	Uveal Melanoma

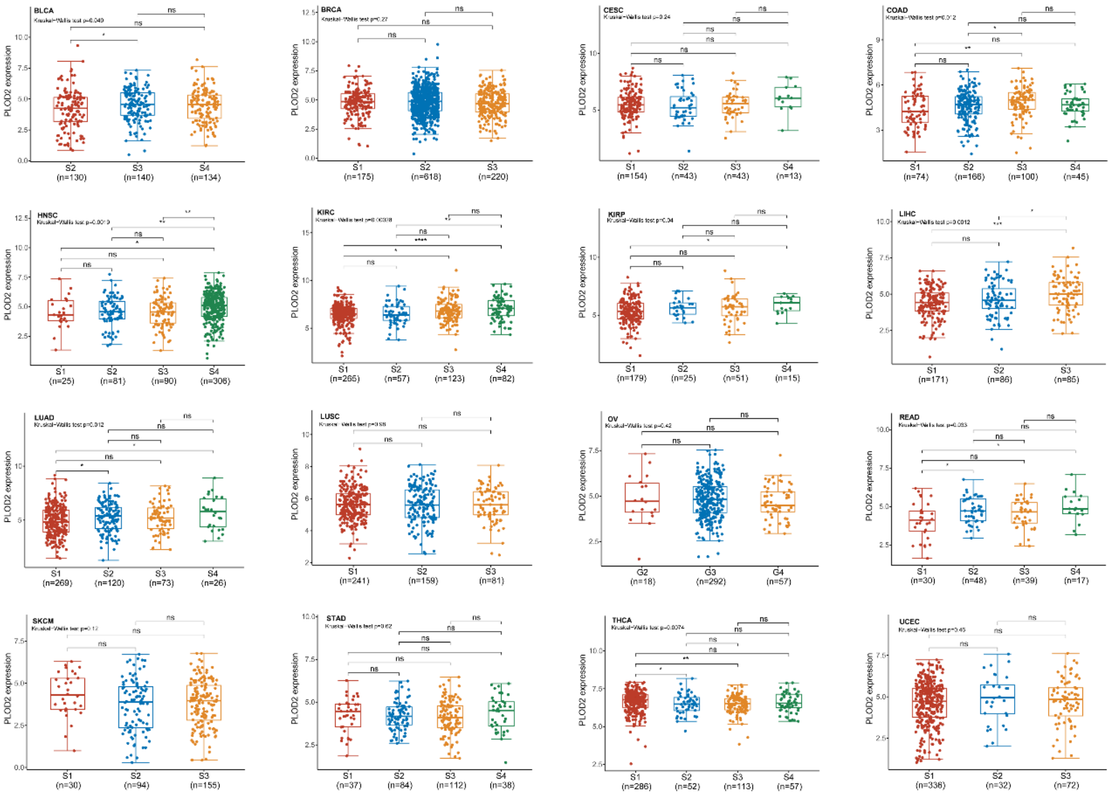

Fig. S1. The box plot shows the association of *PLOD2* expression with pathological stages for different types of cancers.

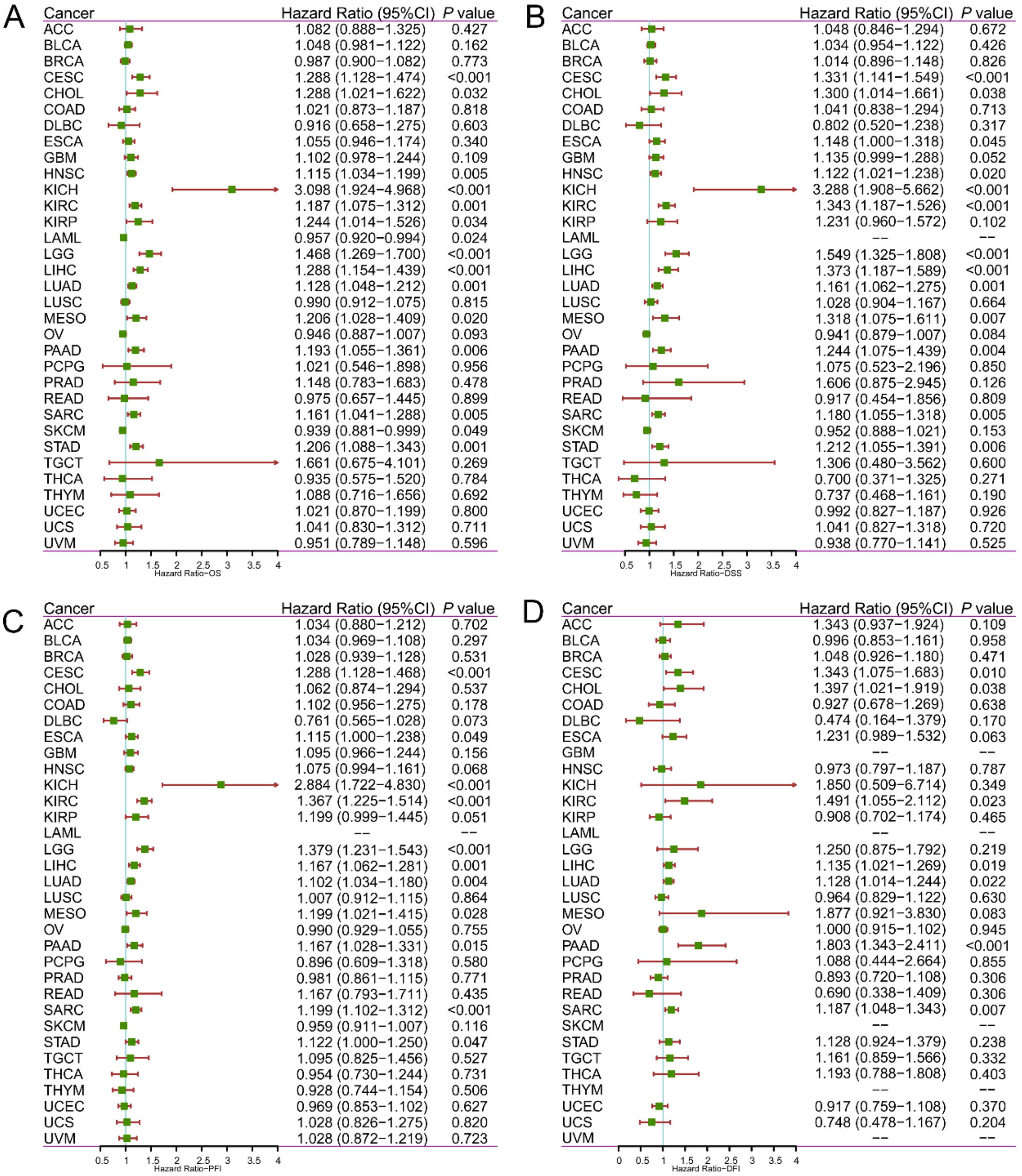

Fig. S2. Association of *PLOD2* expression with patient survival. **(A)** overall survival **(B)** disease-specific survival **(C)** progression-free interval **(D)** disease-free interval.

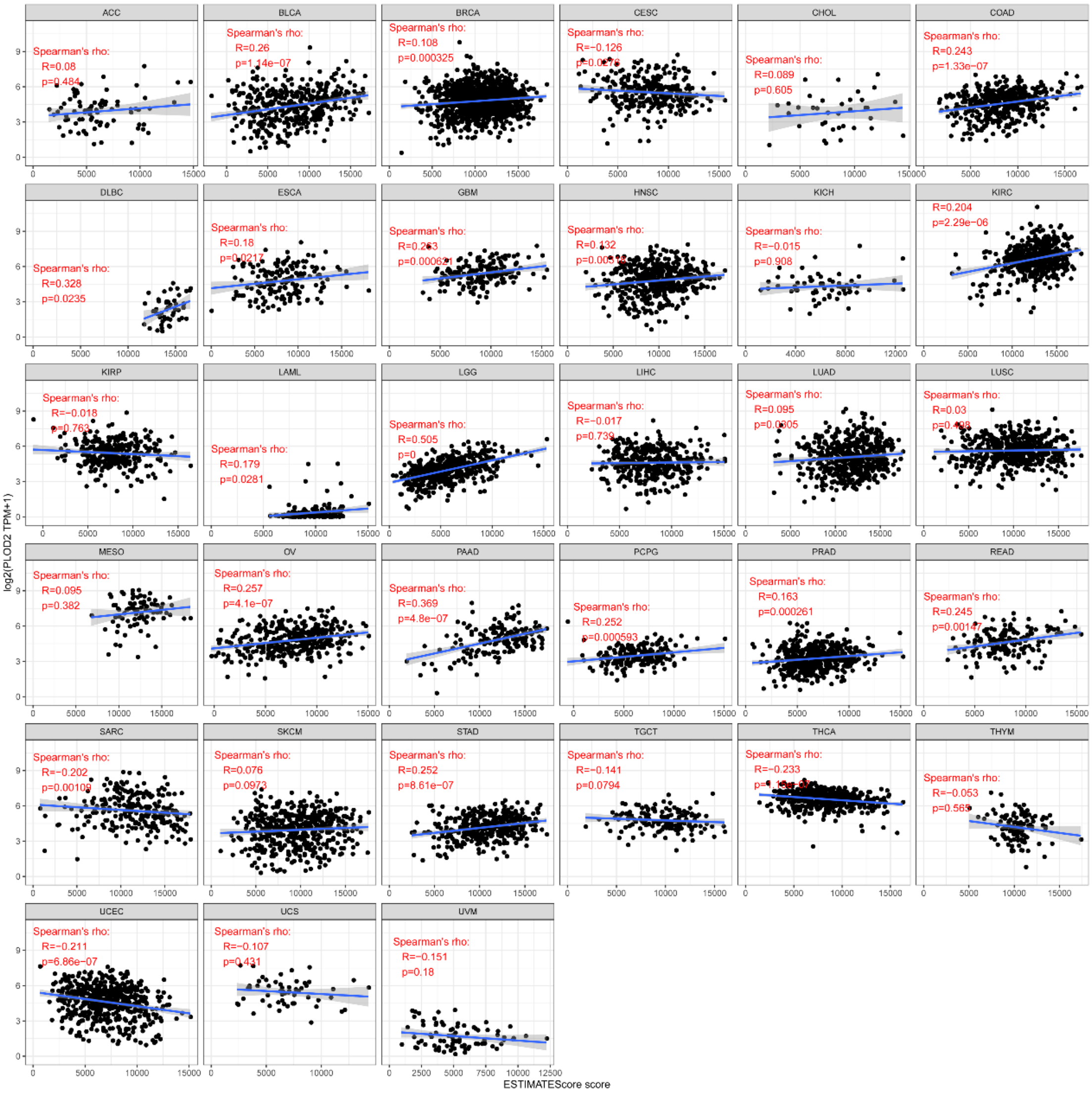

Fig. S3. Relationship between *PLOD2* gene expression and the ESTIMATE Score.

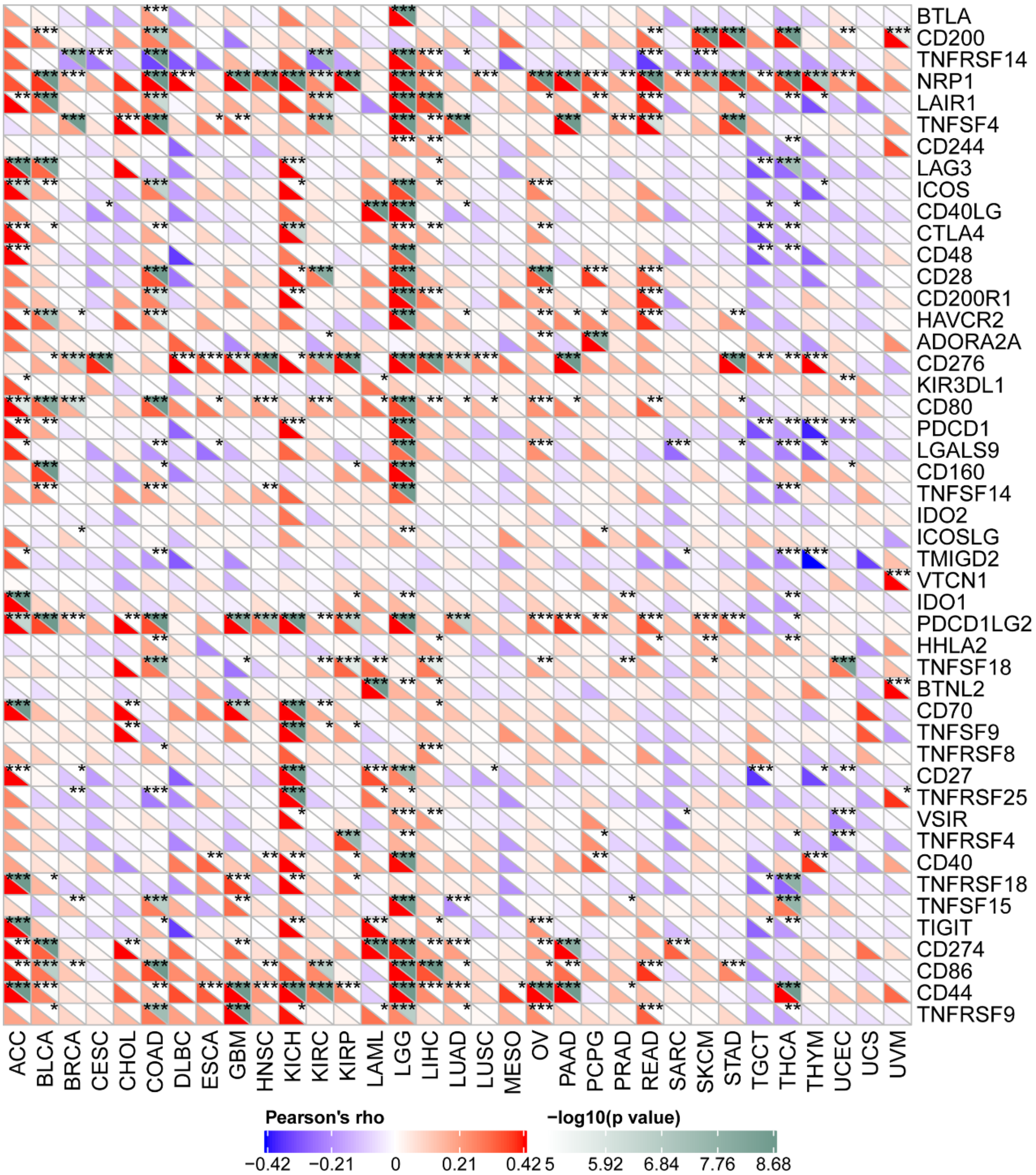

Fig. S4. Heatmap of *PLOD2* gene expression and the immune checkpoint genes. For each pair, the right triangle is colored to represent the P-value; the bottom left is colored to indicate the Pearson's correlation coefficient. *$P < 0.05$; **$P < 0.01$; ***$P < 0.001$.

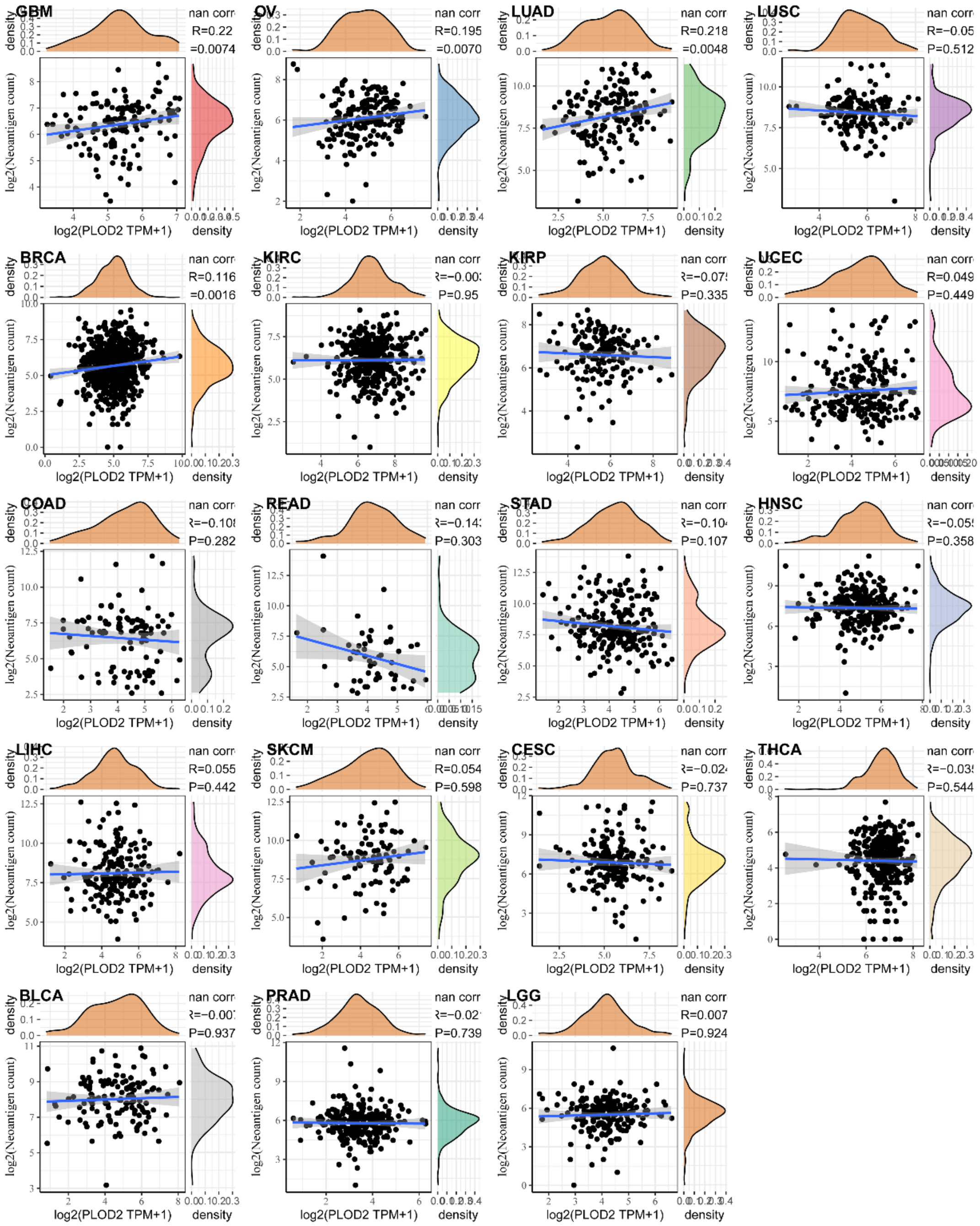

Fig. S5. Relationship between the tumor neoantigen burden and *PLOD2* gene expression in each tumor.

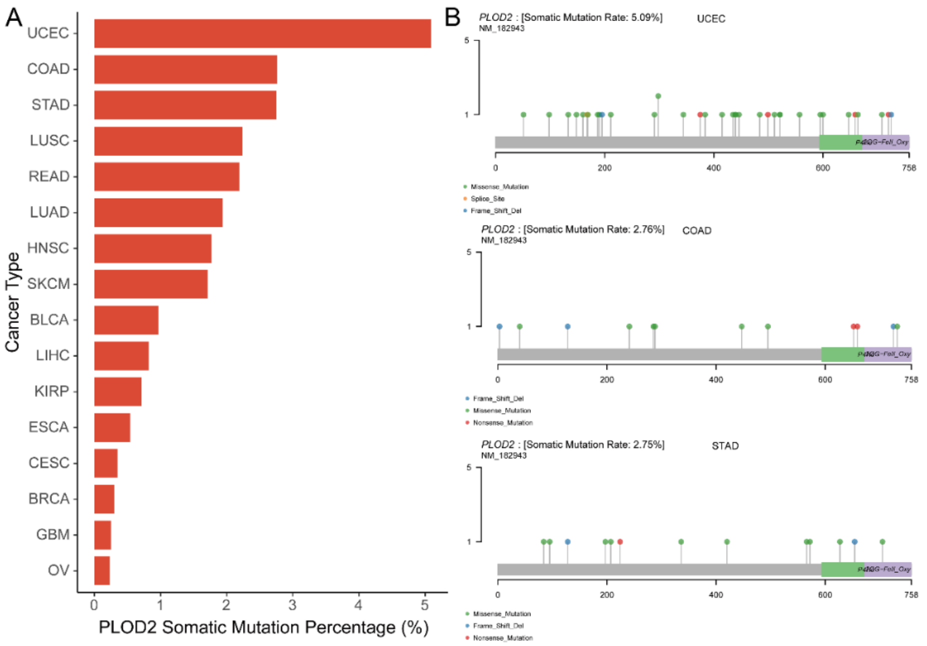

Fig. S6. The genetic alteration status of *PLOD2* in pan-cancer. **(A)**The proportion of *PLOD2* mutations in each tumor. **(B)** Distribution of mutations in the top 3 tumors.

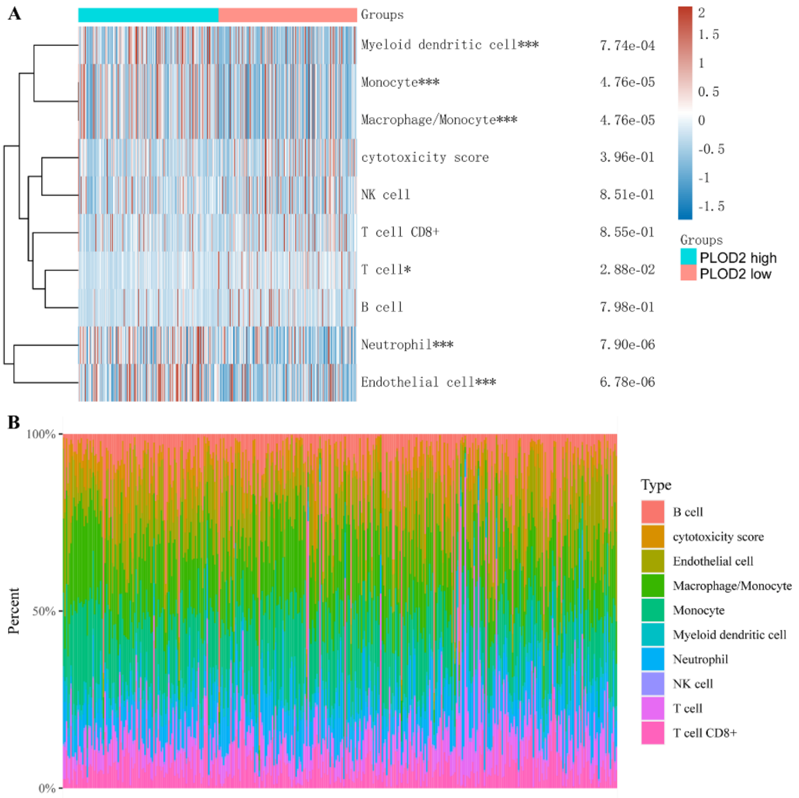

Fig. S7. **(A)** Heatmap of PLOD2 expression and infiltrating immune cells in STAD based on the MCP-Counter deconvolution methods. **(B)** Histogram of PLOD2 expression and infiltrating immune cells in STAD based on the MCP-Counter deconvolution methods.
